# A Multiscale Modeling and Experimental Study on the Tensile Strength of Plain-Woven Composites with Hybrid Bonded–Bolted Joints

**DOI:** 10.3390/polym16142074

**Published:** 2024-07-20

**Authors:** Jianwei Shi, Junwei Zhang, Kou Du, Qiming Guo, Yuliang Hou, Cheng Dong

**Affiliations:** 1 School of Mechanical and Power Engineering, Zhengzhou University, Zhengzhou 450001, China; 2 Inner Mongolia North Heavy Industries Group Co., Ltd., Baotou 014010, China

**Keywords:** finite element analysis, hybrid bonded–bolted (HBB) joints, multiscale modeling, neural networks, deep learning

## Abstract

CFRP hybrid bonded–bolted (HBB) joints combine the advantages of traditional joining methods, namely adhesive bonding, and bolting, to achieve optimal connection performance, making them the most favored connection method. The structural parameters of CFRP HBB joints, including overlap length, bolt-hole spacing, and fit clearance relationships, have a complex impact on connection performance. To enhance the connectivity performance of joint structures, this paper develops a multiscale finite element analysis model to investigate the impact of structural parameters on the strength of CFRP HBB joint structures. Coupled with experimental validation, the study reveals how changes in structural parameters affect the unidirectional tensile failure force of the joints. Building on this, an analytical approach and inverse design methodology for the mechanical properties of CFRP HBB joints based on deep supervised learning algorithms are developed. Neural networks accurately and efficiently predict the performance of joints with unprecedented combinations of parameters, thus expediting the inverse design process. This research combines experimentation and multiscale finite element analysis to explore the unknown relationships between the mechanical properties of CFRP HBB joints and their structural parameters. Furthermore, leveraging DNN neural networks, a rapid calculation method for the mechanical properties of hybrid joints is proposed. The findings lay the groundwork for the broader application and more intricate design of composite materials and their connection structures.

## 1. Introduction

Carbon fiber-reinforced polymers (CFRPs) possess high specific strength, stiffness, corrosion resistance, and excellent design flexibility [1,2,3]. These materials and their joint structures have seen increasing use in aerospace, automotive, wind energy, and daily life products [4,5,6,7,8]. With the rapid development of CFRPs, there is a growing need to enhance the mechanical properties of these materials and their joint structures to meet new performance requirements [9].

Joint structures, which serve as critical load-transfer nodes between CFRP components, are also the most vulnerable areas but are crucial for the overall load-bearing capability and reliability of the assembly [10]. Different joint configurations display distinct mechanical behaviors; therefore, the analysis and design of CFRP joint structures significantly impact their performance [11]. Commonly used mechanical joints include bolting joints and bonding joints. Research has shown that hybrid bolted–bonded joints (HBB) combine the advantages of bolting joints and bonding joints, resulting in improved mechanical performance.

Studies by researchers like Xu Chang [12] have demonstrated that HBB joints offer an average increase in load-bearing capacity of 40.5% and 31.9% compared to adhesive and bolted joints, respectively. Gamdani Farid and colleagues [13] conducted experiments on HBB joints made from laminated and woven boards, finding that these joints enhance mechanical performance by 30% and 70% compared to bolted joints alone. They [14] also observed that HBB joints consistently outperform bolted joints in fatigue tests, with increased bolt numbers further boosting this advantage. Ulus Hasan’s research indicates [15] that HBB joints excel in mechanical performance and energy absorption across all temperatures compared to purely adhesive or bolted joints. Jiang Lanxin’s experiments [16] on dual-bolt HBB joints revealed uneven stress distribution between bolts, leading to earlier damage at the more heavily stressed bolt. However, the inclusion of an adhesive layer significantly improves this stress distribution, thereby enhancing the joint’s strength. Zheng Yanping and team [17] developed a model for a CFRP to titanium alloy HBB joint, finding that dual-bolt configurations are 82.6% stronger than single-bolt setups, and triple-bolt structures are 34.1% stronger than dual-bolt ones. He Boling’s studies [18] showed that aligning composite fibers in the 0° direction significantly increases the stiffness and strength of the joint structure. Li Xin’s experiments [19] confirm the clear advantages of dual-bolt over single-bolt structures, with increases in bolt diameter positively impacting mechanical performance. Because the adhesive layer in HBB structures and the epoxy resin in laminated composites are susceptible to environmental changes [20,21], Delzendehrooy F. and others [22] conducted experimental studies on the degradation of various HBB structures under aging conditions. They found that although increasing the bolt size can enhance the ultimate failure load of the connection structure, structures with larger ratios of width-to-hole diameter and edge-to-hole diameter exhibit more pronounced adhesive failures. Li Xiaoqi and colleagues [23] investigated the impact of material properties and cross-sectional shapes on the mechanical performance of HBB connection structures, observing that bolts only begin to carry significant loads after adhesive damage reaches the bolt holes. Using interference fits, high-modulus and high-strength bolts significantly raise the pre-failure load-bearing capacity of bolts, and using low-modulus, high-strength adhesives is key to enhancing the joint’s mechanical performance and energy absorption.

In multi-bolt structures, variations in bolt load distribution can lead to damage in areas under greater stress, reducing the strength of the joint. Xu Zhi Xiang [24] conducted analyses on the load distribution of hybrid bolted–bonded joints with three bolts using conventional two-dimensional finite element methods, new two-dimensional finite element methods, and three-dimensional finite element methods. It was found that the load distribution on the middle bolt of the joint is less than that on the bolts at the ends, and that lower preload has almost no effect on the load distribution.

Liu Feng Rui and colleagues [25,26] conducted studies on the load distribution of bolted connection structures, discovering that the clearance between bolt holes has a significant impact on the load distribution within bolted structures. Qiu Cheng and colleagues [27] verified the uneven load distribution phenomenon in bolted structures through improved circuit model analysis and experiments, utilizing heuristic algorithm-based optimization methods to find gap fit and preload parameter schemes that ensure even load distribution. McCarthy MA’s research [28] confirms that even the use of very small fit clearances can significantly alter the load distribution within HBB joints, consistent with previous research findings.

Designing HBB joints to achieve unprecedented performance levels is crucial for the design and application of CFRPs [29]. The complexity of HBB joint structures requires a substantial design space [30]. Furthermore, as design precision and demands increase, traditional design methods centered around experimental observation, theoretical modeling, and numerical simulation are no longer sufficient to meet current requirements. This indicates a clear need for new design methodologies [31]. Therefore, this paper develops a supervised learning method for joint design, based on experimental and finite element simulation studies of the mechanical performance of HBB joints.

## 2. Experimental Section

### 2.1. Material Parameters

This experiment employs T300/7901 carbon fiber-reinforced polymer (CFRP) laminates to fabricate hybrid bolted–bonded (HBB) joint specimens. The selected laminate model has a total thickness of 0.24 mm with six layers, each oriented at [0/90] degrees, as detailed in Table 1 [32]. The adhesive used in the bonding area of the HBB joints is the Araldite 2015 epoxy adhesive, as detailed in Table 2. For the clamping ends of the HBB joints, 5182 aluminum alloy plates are selected as shims. These plates have the same thickness as the T300/7901 composite material, ensuring the tension direction remains horizontal during tensile testing.

### 2.2. Specimen Fabrication

**Figure 1:**
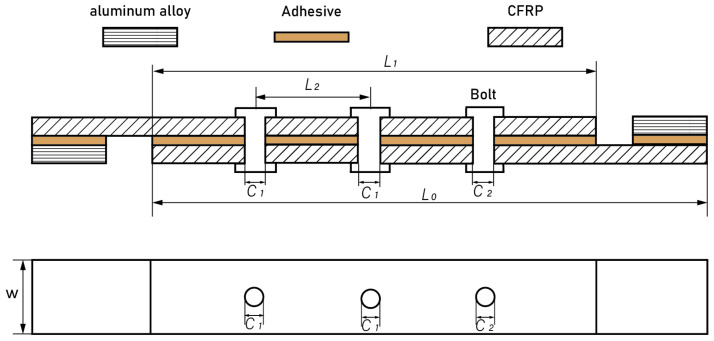
A structural schematic diagram of a CFRP hybrid bonded–bolted joint.

This paper follows the experimental standard ASTM 5961 [33] for the fabrication and testing of CFRP HBB joints. Figure 1 shows a schematic of the HBB joint. The total length of the structure is *L*_0_ = 190 mm, and its width is *W* = 30 mm, with an overlap region of *L*_1_ = 90 mm. The bolt holes are located on the centerline of the width of the specimen, with both holes having diameters of *C*_1_ = *C*_2_ = 5 mm. This study consistently uses a bolt diameter of 5 mm, and the adhesive layer thickness is maintained at 0.1 mm throughout. To reduce the impact of bending stress on the joint during testing, aluminum alloy shims measuring 50 × 30 mm^2^ are bonded to both clamping ends of the joint, ensuring alignment with the fixtures of the testing apparatus.

High-pressure water jet cutting is used to trim the composite laminate panels to the target dimensions. The surfaces of the bonding areas are then sanded along the fiber direction to enhance the roughness of the laminate surface, which increases the contact area between the laminate and the adhesive, thereby improving the adhesion in the joint bonding area. The surfaces are cleaned with acetone after sanding. Araldite 2015 epoxy adhesive is applied uniformly across the bonding area. Bolts are then installed using an electronic torque wrench to ensure that there is no preload on the bolts. After the adhesive application, the specimens are secured with dovetail clamps and left to rest in a cool place for 12 h. After standing, the specimens are placed in a vacuum oven set at 120 °C and heat-cured for 2 h [34].

### 2.3. Experimental Methodology

Four sets of control experiments are designed to study the impact of structural parameter variations on the mechanical performance of CFRP hybrid bolted–bonded joints. Each set involves altering a single structural parameter—overlap length (*L*_1_), the spacing between bolt holes (*L*_2_), the diameter of the bolts at both ends (*C*_1_), and the diameter of the middle bolt hole (*C*_2_). Each experiment set is replicated three times, with the specimen parameters detailed in Table 3.

When varying the structural parameters, the position of the central bolt remains unchanged, and the bolts at both ends are always maintained symmetrically. Changes in the spacing between bolt holes are achieved by adjusting the positions of the bolts at the ends. The gap fit relationship is managed by changing the bolt-hole diameters while keeping the bolt sizes constant. The experiments utilize a WDW-300 tensile testing machine from the Changchun Kexin Company in Changchun,China. During the experiments, one end of the specimen is completely fixed, while the other end is stretched at a uniform speed of v = 2 mm/min. The tensile force and displacement data are collected from the load cell sensor of the universal tensile testing machine.

### 2.4. Experimental Results Analysis

To ensure the accuracy of the experimental results, the median of three test groups was used for the analysis of the ultimate failure load. Damage failure analysis was carried out using the Keyence Digital Microscope VHX-6000 in Borken, Germany hyper-depth of field equipment to capture local damage areas in the CFRP HBB joints.

#### 2.4.1. Effect of Overlap Length (*L*_1_) on Joint Mechanical Performance

**Figure 2:**
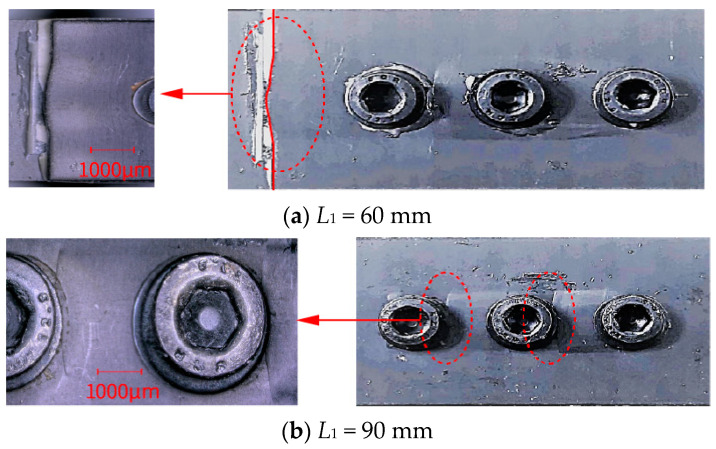
Comparative analysis of impact of overlap length (*L*_1_) on joint mechanical performance: (**a**) primary failure region when overlap length (*L*_1_) is 60 mm, and (**b**) primary failure region when *L*_1_ is 90 mm.

Figure 2a,b show a comparative magnified view of the failure areas for joints with *L*_1_ = 60 mm and *L*_1_ = 90 mm, respectively. When *L*_1_ is 60 mm, the end of the overlap area is closer to the bolt, leading to lower load-bearing performance and damage due to compression by the bolt, making it the primary failure area. With an *L*_1_ of 90 mm, the end of the laminate overlap area is further from the bolts, enhancing its load-bearing performance; the main failure at this length is damage due to compression by the three bolts.

The median ultimate failure load for HBB joints with an *L*_1_ of 90 mm is 33.4 KN, compared to 25.48 KN for an *L*_1_ of 60 mm, representing an increase of 29.9%. Therefore, increasing the overlap length can lead to a more even distribution of load among the three bolts, which significantly enhances the ultimate failure strength of the joint. This finding suggests that by optimizing the overlap length, the mechanical integrity and load distribution of HBB joints can be substantially enhanced, leading to improved performance under tensile stress.

#### 2.4.2. Effect of Bolt-Hole Spacing (*L*_2_) on Joint Mechanical Performance 

**Figure 3:**
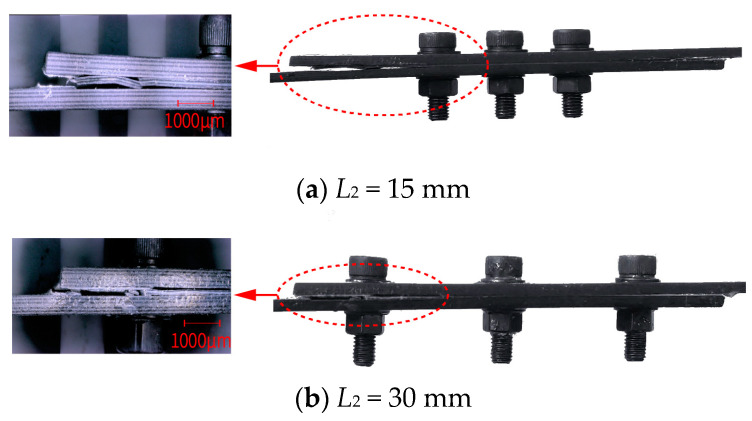
(**a**) Primary failure region when bolt-hole spacing (*L*_2_) is 15 mm, and (**b**) primary failure region when *L*_2_ is 30 mm.

Figure 3a,b show a magnified view of the failure areas for joints with *L*_2_ = 15 mm and *L*_2_ = 30 mm, respectively. When *L*_2_ is 15 mm, the ends of the overlap area are further from the bolts, resulting in a higher load-bearing performance; thus, the primary failure occurs outside the bolts at the ends of the overlap area, where delamination damage due to normal forces is evident. Conversely, when *L*_2_ is 30 mm, the ends of the overlap area are closer to the bolts, leading to lower load-bearing performance, with the main failure being delamination damage near the bolts caused by normal forces. The median ultimate failure load for joints with *L*_2_ = 15 mm is 33.4 KN, compared to 28.99 KN for *L*_2_ = 30 mm, an increase of 15%. Therefore, reducing the bolt-hole spacing enhances the adhesive performance at both ends of the overlap area, effectively reducing damage due to normal forces, and thereby improving the mechanical performance of the HBB joints.

#### 2.4.3. Effect of Fit Clearance on Joint Mechanical Performance

**Figure 4:**
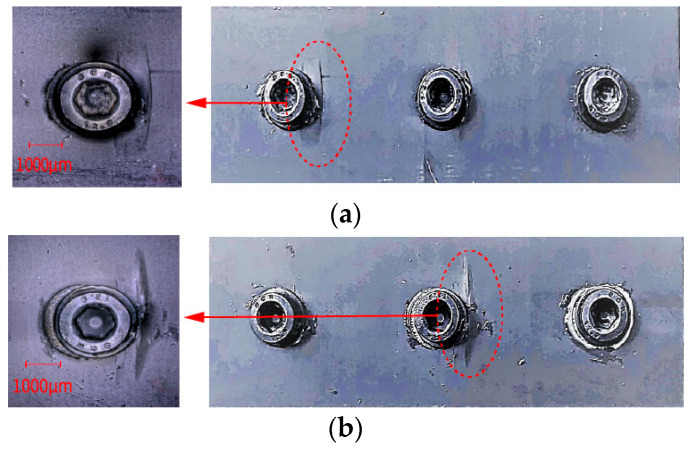
(**a**) The primary failure region when all three bolts have a normal fit, and (**b**) the primary failure region when the two end bolts have a clearance fit.

To explore the impact of gap fit on joint load distribution, the diameters of the bolt holes at both ends were increased to create a clearance fit for the end bolts. Figure 4a,b compare the failure areas for bolt-hole diameters of *C*_1_ = 5 mm and *C*_1_ = 5.3 mm. When *C*_1_ is 5 mm, the load distribution on the end bolts is greater than on the middle bolt, and the main failure area is near the end bolts where the laminate is damaged by bolt compression. When *C*_1_ is 5.3 mm, the clearance fit at the ends reduces the load distribution during tensile testing, and the main failure area shifts to near the middle bolt where damage occurs due to bolt compression. The median ultimate failure load for joints with a bolt-hole diameter of 5.3 mm at the ends is 30.82 KN, compared to 28.99 KN when the diameter is 5 mm, an increase of 6.9%. Thus, applying a gap fit reduces the load distribution on the end bolts and significantly increases it on the middle bolt, thereby enhancing the mechanical performance of the HBB joints. This suggests that strategic manipulation of the gap fit can effectively redistribute loads within a joint, optimizing performance and reducing localized damage risks.

## 3. Finite Element Analysis

### 3.1. Model Establishment

A three-dimensional finite element model of the CFRP hybrid bolted–bonded (HBB) joint structure was established in ABAQUS. This model utilized solid element modeling techniques, with the laminate employing the three-dimensional Hashin failure criterion as the damage criterion and the adhesive layer using a cohesive model for damage assessment. This setup effectively simulates the tensile process of the HBB joint and achieves optimal simulation results.

Each laminate in the model is subdivided into six layers according to its thickness, with each layer representing a [0/90] laminate and each having a thickness of 0.4 mm. Given that the strength of the bolts used in the experiments far exceeds that of the laminate, the three bolts in the model are represented using homogeneous, isotropic material properties without damage. During the numerical tensile analysis, the bolts undergo deformation but do not experience damage. The adhesive behavior of the model uses a damageable adhesive contact configuration, while other contact interactions are set as hard contacts. A penalty function friction coefficient of 0.15 is applied to account for the effects of friction and gap fit relationships during the numerical tensile analysis.

The model employs linearly reduced integration of eight-node solid C3D8R elements for meshing, with refinement in the mesh at the areas around the bolts and the laminate to ensure accuracy in these critical regions. Detailed modeling specifics can be found in the Reference [35].

### 3.2. Simulation Results Validation 

#### 3.2.1. The Impact of Overlap Length (*L*_1_) on the Mechanical Performance of Joints

**Figure 5:**
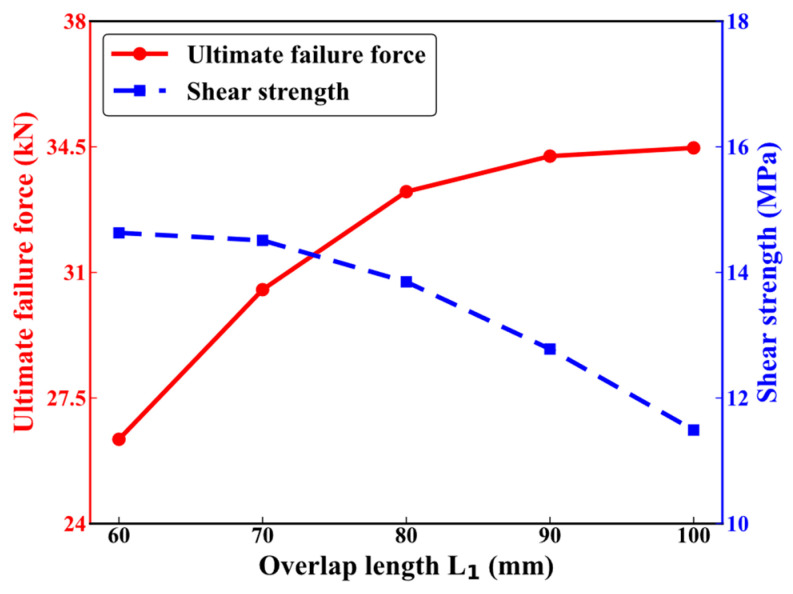
Mechanical performance of hybrid bolted–bonded joints with varying overlap lengths (*L*_1_).

Numerical tensile analyses are conducted on CFRP HBB joints with overlap lengths ranging from 60 mm to 100 mm. Figure 5 illustrates the impact of varying overlap lengths on the performance of the HBB joint. The ultimate failure loads of the HBB joints, as indicated by the red line in the figure, are 26.31 KN, 30.52 KN, 33.25 KN, 34.13 KN, and 34.47 KN. The increase in overlap length enlarges the bonded area of the joint, thus enhancing the ultimate failure force as the overlap length increases, although the rate of increase gradually slows down. To comprehensively analyze the impact of overlap length on joint connectivity performance, the formula for calculating the shear strength of the CFRP HBB joint is as follows:

(1) $$\tau =\frac{F_{f}}{L\times W}$$

where τ represents the shear strength, $F_{f}$ is the ultimate failure load, and L and W are the length and width of the overlap area, respectively.

In Figure 5, the blue dashed line represents the shear strength of the HBB joints, with corresponding values of 14.63 MPa, 14.51 MPa, 13.85 MPa, 12.64 MPa, and 11.49 MPa. The shear strength decreases with increasing overlap length, which is opposite to the trend observed with the ultimate failure force. Properly designing the overlap length of the joint can reduce shear stress, enhance ultimate failure load, and improve mechanical performance.

**Figure 6:**
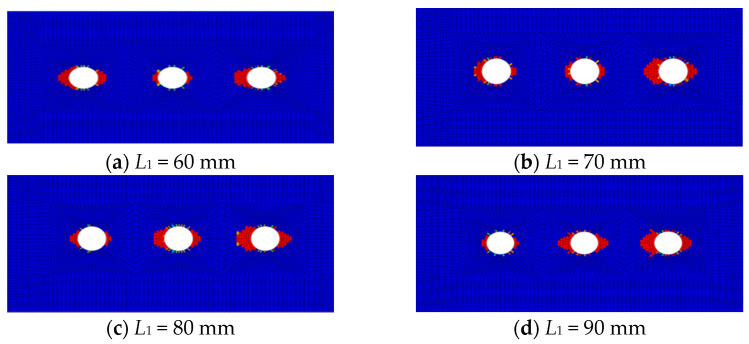
Fiber compression damage in hybrid bolted–bonded (HBB) joints at various overlap lengths.

Figure 6 displays the fiber compression damage in the overlap area of the CFRP HBB joints at different overlap lengths. The figure reveals that at shorter overlap lengths, damage near the bolt holes at the ends is more pronounced compared to the damage near the middle bolt hole. As the overlap length increases, the damage near the middle bolt hole becomes significantly more noticeable, consistent with experimental results.

#### 3.2.2. The Influence of Bolt-Hole Spacing on the Mechanical Performance of Joints

**Figure 7:**
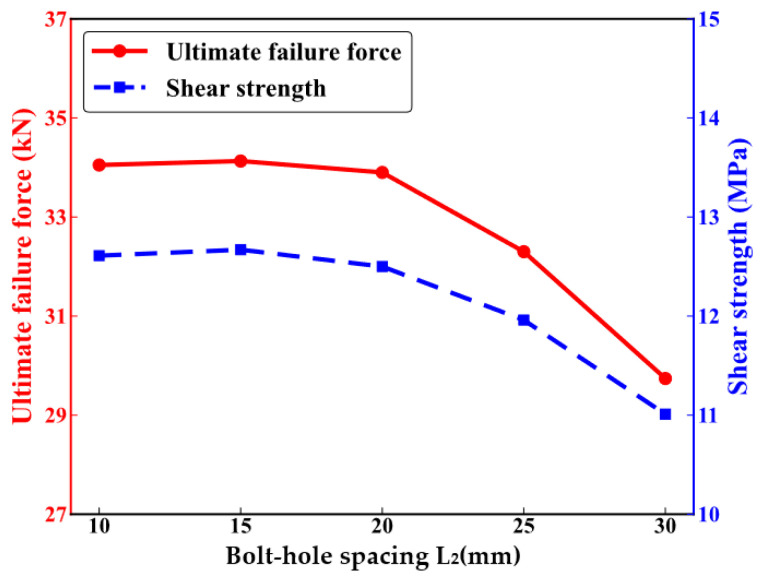
Mechanical performance of hybrid bolted–bonded joints with varying bolt-hole spacings.

Numerical tensile analyses are carried out on CFRP HBB joint structures with bolt-hole spacings ranging from 10 mm to 30 mm to study the impact of bolt-hole spacing on the mechanical performance of the joints. The ultimate failure loads of the joints, as indicated by the red line in Figure 7, are 34.05 KN, 34.13 KN, 33.9 KN, 32.3 KN, and 29.74 KN. The ultimate failure load decreases gradually and then more sharply as the bolt-hole spacing increases. The shear strength of the HBB joints, represented by the blue dashed line in Figure 7, shows values of 12.61 MPa, 12.64 MPa, 12.5 MPa, 11.96 MPa, and 11.01 MPa. The trend in shear strength mirrors that of the ultimate failure load, where it increases initially and then decreases as the bolt-hole spacing increases. Smaller bolt-hole spacings slightly reduce the shear performance of the CFRP HBB joints but can significantly enhance the ultimate failure load.

**Figure 8:**
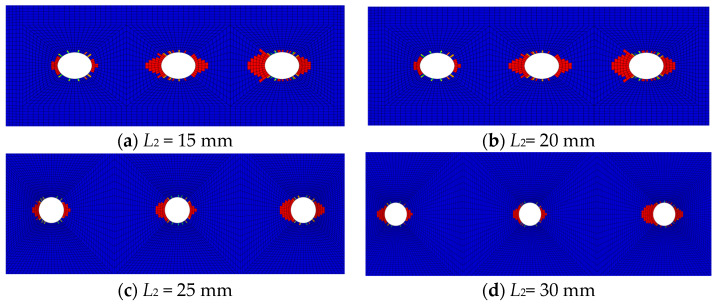
Fiber compression damage in HBB joints at varying bolt-hole spacings.

Figure 8 displays the fiber compression damage in the overlap areas of the CFRP HBB joints at different bolt-hole spacings. At smaller spacings, the damage near the bolt holes on the inner side of the stretch direction is comparable to that near the middle bolt hole. As the bolt-hole spacing increases, the damage near the middle bolt hole significantly decreases, suggesting that the load borne by the middle bolt is gradually reducing. Therefore, decreasing the bolt-hole spacing is beneficial for increasing the load distribution on the middle bolt, aligning with experimental results.

#### 3.2.3. The Impact of the Fit Clearance Relationship on the Mechanical Performance of Joints

**Figure 9:**
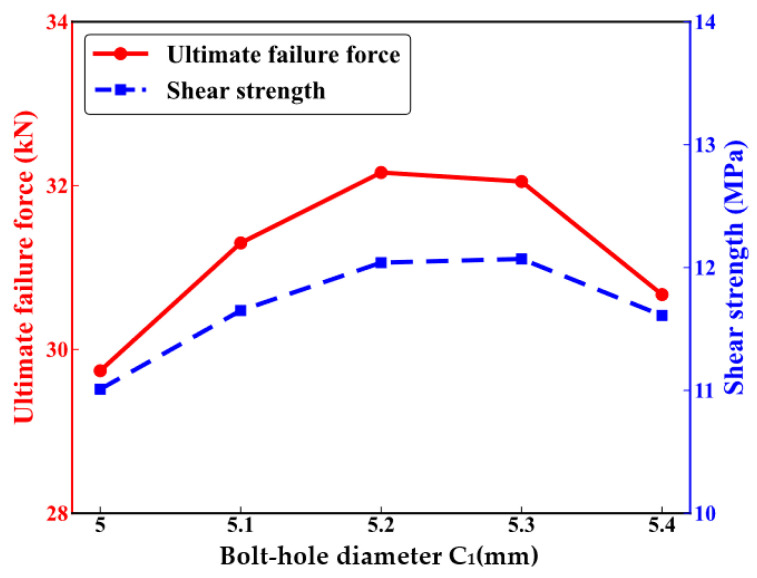
Mechanical performance of hybrid bolted–bonded joints with varying diameters of bolt holes at the ends.

Further numerical tensile analyses were conducted on CFRP HBB joint structures with gap fits ranging from 5.0 mm to 5.4 mm to study the impact of gap fit relationships on the mechanical performance of the joints. Due to the symmetric nature of the numerical model, the gap fit relationships were analyzed separately for the bolts at both ends and the middle bolt. The bolt sizes remained constant while the bolt-hole diameters were increased to achieve the gap fit. Figure 9 shows the impact of the end bolt-hole diameters on the mechanical performance of the CFRP HBB joints. The ultimate failure load, depicted by the red line, exhibits values of 29.74 KN, 31.3 KN, 32.16 KN, 32.05 KN, and 30.67 KN as the end bolt-hole diameters increase. The shear strength, shown by the blue dashed line, displays values of 11.01 MPa, 11.65 MPa, 12.04 MPa, 12.07 MPa, and 11.61 MPa, following the same trend as the ultimate failure load, where it increases initially and then decreases with the increase in end bolt-hole diameters. 

**Figure 10:**
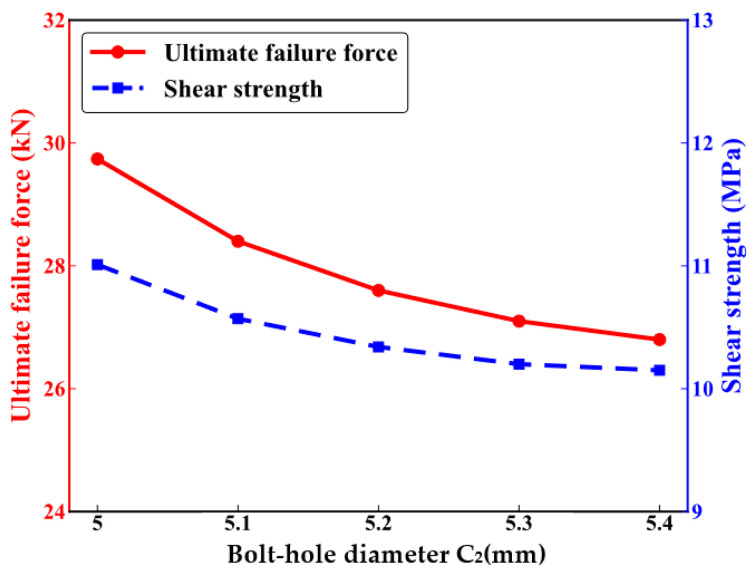
The mechanical performance of hybrid bolted–bonded joints with varying diameters of the central bolt hole.

Figure 10 shows the impact of the middle bolt-hole diameter on the mechanical performance of CFRP HBB joints. The ultimate failure loads, indicated by the red line, are 29.74 KN, 28.41 KN, 27.67 KN, 27.12 KN, and 26.87 KN. The ultimate failure force gradually decreases as the diameter of the central bolt hole increases, although the trend slows down over time. The blue dashed line in the figure represents the shear strength of the HBB joints, with corresponding values of 11.01 MPa, 10.57 MPa, 10.34 MPa, 10.20 MPa, and 10.15 MPa. Shear strength also decreases with the increasing diameter of the middle bolt hole, following the same trend as the ultimate failure load.

**Figure 11:**
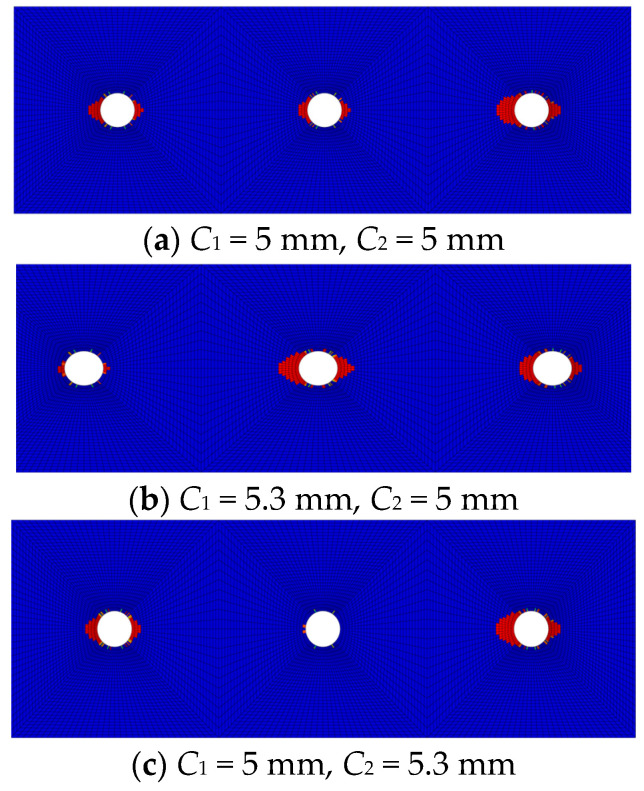
Fiber compression damage in HBB joints under different gap fits.

Figure 11 displays fiber compression damage in the overlap areas of CFRP HBB joints under different gap fit relationships. Compared to joints without gap fits, those with gap fits at the bolts at both ends show significantly more damage near the middle bolt hole, indicating an increased load distribution on the middle bolt. When the middle bolt is under a gap fit, there is virtually no damage near the middle bolt hole, suggesting a very low load distribution on the middle bolt. Therefore, decreasing the bolt-hole spacing is beneficial for increasing the load distribution on the middle bolt, consistent with experimental results.

The modeling approach presented has been proven to accurately and effectively calculate the ultimate failure load of CFRP HBB joint structures. Consequently, the dataset labels required for training the model in Section 4 are calculated using this finite element model.

## 4. Analysis of Mechanical Properties of HBB Joints Based on Artificial Intelligence 

### 4.1. Design of Deep Neural Network (DNN) Calculator

Using numerical simulations to study numerous scenarios is complex and time-consuming, making the execution of structural parameter design processes highly challenging. Therefore, there is a need for advanced universal design methods. The rapid development of supervised learning provides potential solutions for the swift design of mechanical problems. The literature reference [36] discusses the trends, methods, and challenges in traffic prediction using deep neural networks, providing a direction for advancement in this field. Similarly, more researchers are applying computational methods to various engineering and medical problems [37,38,39,40,41]. This paper applies supervised learning algorithms to the field of mechanics, developing a new method for designing CFRP hybrid bolted–bonded (HBB) joint structures based on supervised learning.

In the present study, tensile experiments were designed for HBB joints with different structural parameters, and the quasi-static tensile strength of CFRP HBB joints was tested. The accuracy of the numerical model was validated by comparing experimental results with numerical outcomes. Both experimental measurements and numerical simulations were used as testing datasets, demonstrating that the finite element model can be utilized to compute the mechanical properties of the dataset.

**Figure 12:**
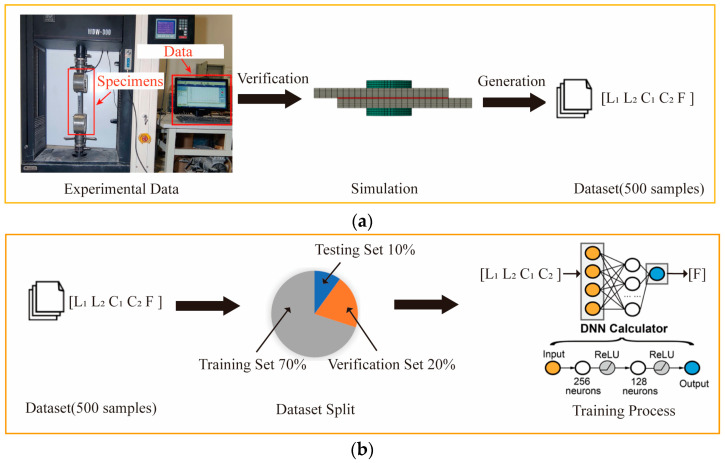
(**a**) Dataset generation process for HBB joint analysis; (**b**) schematic of DNN calculator structure for HBB joint analysis.

The DNN (deep neural network) calculator is a deep neural network that takes the structural array [*L*_1_, *L*_2_, *C*_1_, *C*_2_] of CFRP HBB joints as input and outputs mechanical performance. The dataset generation process for training the DNN calculator, as shown in Figure 12a, uses a validated finite element model to accurately simulate the joint’s ultimate failure load. A dataset of 500 data sets was constructed within the design space using a uniform sampling method, with corresponding failure loads calculated using finite element analysis [35]. The DNN is trained using the labeled dataset to develop a highly accurate DNN calculator. After sufficient training, the DNN calculator can accurately predict the ultimate failure loads for unseen parameter combinations without the need for additional numerical simulations, greatly accelerating the design process.

The designed DNN calculator structure, as shown in Figure 12b, comprises two hidden layers with 256 and 128 neurons, respectively. The ReLU (Rectified Linear Unit) activation function is used for the hidden layers to introduce nonlinearity, endowing the DNN calculator with a robust nonlinear fitting capability. During the training process, the dataset is partitioned into training, validation, and test sets, with respective proportions of 70%, 20%, and 10%. The optimization process utilizes the Adam optimizer [42], an effective stochastic gradient descent backpropagation method, along with a learning rate decay strategy to enhance the computational accuracy of the DNN calculator [43]. Additionally, to prevent overfitting in the predictive model, regularization techniques such as Dropout [44] and early stopping are implemented effectively to curb overfitting behaviors [45]. 

### 4.2. Performance Evaluation of DNN Calculator

**Figure 13:**
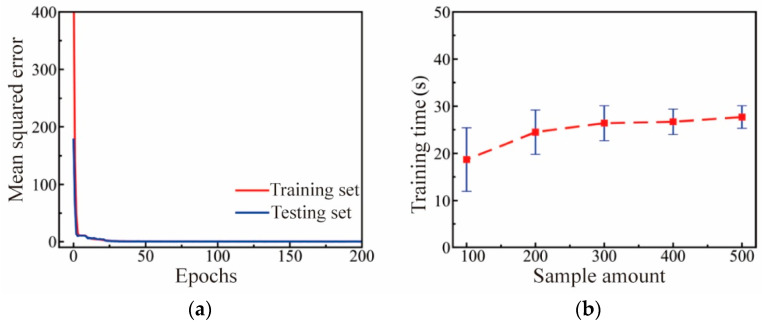
Regression performance evaluation of DNN calculator: (**a**) Variation of mean squared error during iterations when sample amount is 500; (**b**) influence of sample amount on training time.

The number of sample datasets used to train the DNN calculator significantly impacts the model’s performance. Therefore, this paper randomly selects 100, 200, 300, 400, and 500 sets from a total of 500 samples to study the effect of sample size on the regression performance of the DNN calculator. The results are shown in Figure 13. As illustrated in Figure 13a, with a sample size of 500, the mean squared error for both the training and validation sets decreases rapidly during the training process and converges after 25 epochs. Figure 13b displays the influence of sample size on the training time of the DNN calculator. It can be observed that the difference in required training time is negligible across sample sizes, and the fluctuation in training time decreases with larger sample sizes, indicating that increasing the number of samples enhances model stability.

Table 4 shows the impact of sample size on prediction accuracy. It can be observed that when the sample size is less than 300, the training set accuracy of the DNN calculator is significantly greater than that of the test set, showing a slight advantage. Increasing the number of samples is beneficial for improving the predictive accuracy of the DNN calculator. When the sample size exceeds 400, both the training set and test set prediction accuracies of the DNN approach 1. Therefore, in this study, a sample size of 500 is considered sufficient.

As shown in Table 5, the regression error distribution between the calculated values and the true values of the ultimate failure force by the DNN calculator is presented. It can be seen that in both the training set and the test set, over 75% of the data have an error of less than 1%. Moreover, the error distribution on the untrained test set is very similar to that on the training set, demonstrating that the DNN calculator possesses high generalization capabilities in predicting the ultimate failure force of joints.

### 4.3. Utilization of DNN Calculator for Analyzing Influence Relations in CFRP HBB Joints

**Figure 14:**
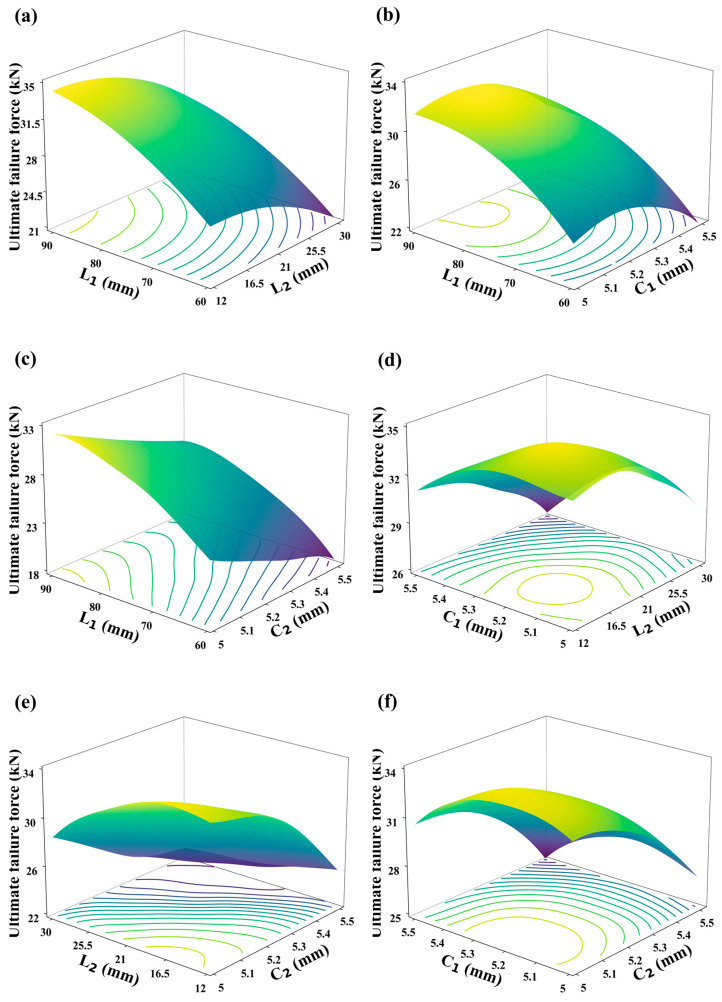
Relationships between joint parameters and ultimate failure load in hybrid bolted–bonded (HBB) joints: (**a**) overlap length (*L*_1_) and bolt-hole spacing (*L*_2_); (**b**) overlap length (*L*_1_) and end bolt-hole diameter (*C*_1_); (**c**) overlap length (*L*_1_) and middle bolt-hole diameter (*C*_2_); (**d**) bolt-hole spacing (*L*_2_) and end bolt-hole diameter (*C*_1_); (**e**) bolt-hole spacing (*L*_2_) and middle bolt-hole diameter (*C*_2_); and (**f**) end bolt-hole diameter (*C*_1_) and middle bolt-hole diameter (*C*_2_).

The trained DNN calculator not only accurately and rapidly converts structural parameters into mechanical performance but also effectively maps the complex relationships between these parameters and performance. As illustrated in Figure 14, the DNN calculator analyzes the relationships between the structural parameters of hybrid bolted–bonded joint configurations and their mechanical performance, with contour lines displayed at the bottom of the graph. When analyzing two parameters, the other two are held constant. From Figure 14a–c, it is evident that the overlap length (*L*_1_) always maintains a positive correlation with the joint’s mechanical performance. However, as the overlap length increases, the rate of improvement in mechanical performance slows down, indicating a plateau effect. Figure 14a,d,e show that as the bolt-hole spacing (*L*_2_) increases, the mechanical performance first increases and then decreases, peaking around a bolt-hole spacing of 15 mm. Figure 14b,d,e reveal that with increasing diameters of the end bolt holes (*C*_1_), mechanical performance first increases and then decreases, with an optimal performance near a diameter of 5.2 mm. From Figure 14c,e,f, it is observed that the mechanical performance decreases with increasing diameter of the middle bolt hole (*C*_2_). These trends suggest that the optimal parameter combinations designed by deep learning offer the best mechanical performance. Additionally, from the range of impacts shown in Figure 14, it is clear that the overlap length has the greatest influence on joint performance, followed by bolt-hole spacing, and finally the diameters of the end and middle bolt holes. The contour lines in Figure 14 indicate that when the joint’s mechanical performance is at its peak, it often coincides with a broad range of contour lines, highlighting significant interdependencies between parameters and making it difficult to intuitively pinpoint the optimal combination. Therefore, the use of efficient and accurate inverse design methods becomes particularly important in the design of structural parameters.

**Figure 15:**
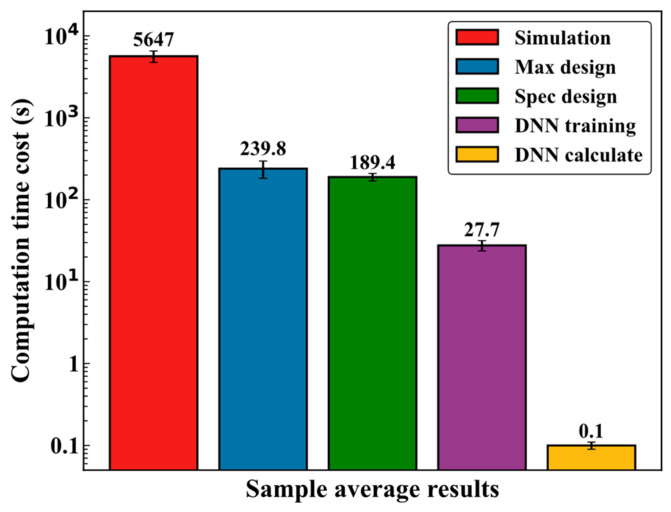
Time cost comparison of different computational design methods for HBB Joints.

In application, the DNN calculator not only demonstrates accuracy but also offers significant advantages in terms of time cost. As shown in Figure 15, the time costs for various methods are compared. Finite element simulations take 5647 ± 1367 s to calculate the mechanical performance of the model, while designs to maximize and specify mechanical performance take 239.8 ± 57.3 s and 189.4 ± 27 s, respectively. DNN training and forward calculation take only 27.7 ± 6.7 s and 0.1 ± 0.01 s, respectively. The results indicate that this method can save a significant amount of time, requiring only 7.5% of the time needed for finite element analysis to meet numerous design requirements.

## 5. Conclusions

This paper focuses on three main aspects of research concerning the mechanical performance analysis and design of CFRP hybrid bolted–bonded (HBB) joint structures: experimental analysis of the mechanical performance of CFRP HBB joints; establishment of a numerical analysis model for CFRP HBB joints; and development of supervised learning methods to analyze and design the mechanical performance of CFRP HBB joints. Experimental results indicate that reducing the bolt-hole spacing, increasing the overlap length, and optimizing the fit clearance all enhance the ultimate failure force of the joint, with the overlap length having the greatest impact on the ultimate failure force. Comparisons of damage failure demonstrated that gap fit has a profound effect on load distribution. A deep neural network (DNN) calculator based on supervised learning was developed and proved efficient in accurately computing the performance of CFRP HBB joints. This tool not only precisely predicts the joint performance for untested parameter combinations but also provides insights into the effects of different parameters on joint performance, greatly accelerating the reverse design process. Additionally, the study validated the efficiency and reliability of a supervised learning framework in exploring optimal design solutions for CFRP HBB joints at a reduced computational cost. The comprehensive analysis of how structural parameters influence the performance of CFRP HBB joints offers essential insights for their development and utilization, highlighting the considerable capabilities of sophisticated computational techniques to enhance the design of joints. The model and experiments presented in this paper can be used as a foundation for further research into the causes of uneven load distribution at joints, to discover more factors that influence load distribution, and to elucidate the patterns by which load distribution affects the performance of various joint connections. Building on the design framework outlined in this paper, advanced design methodologies can be explored, including the implementation of multi-output reinforcement learning design techniques. By leveraging the robust extensibility of neural network models, these techniques can be applied to more complex design challenges to address a wider array of design requirements.

## Figures and Tables

**Table 1:** Material properties of the T300/7901 prepreg in the 0° direction.

Elastic Properties	Value	Damage Properties	Value
Longitudinal Elasticity Modulus, $E_{11}/\mathrm{GPa}$	138	$Tensile\;Strength,\;X_{t}/\mathrm{GPa}$	2
Transverse Elasticity Modulus, $E_{22}/\mathrm{GPa}$	11	$Compressive\;Strength,\;X_{c}/\mathrm{GPa}$	1.15
Normal Modulus of Elasticity, $E_{33}/\mathrm{GPa}$	11	$Tensile\;Strength,\;Y_{t}/\mathrm{GPa}$	0.06
$Poisson’s\;Ratio,\;v_{12}$	0.28	$Compressive\;Strength,\;Y_{c}/\mathrm{GPa}$	0.152
$Poisson’s\;Ratio,\;v_{13}$	0.28	$Normal\;Tensile\;Strength,\;S_{L}/\mathrm{GPa}$	0.075
$Poisson’s\;Ratio,\;v_{23}$	0.4	$Normal\;Compressive\;Strength,\;S_{T}/\mathrm{GPa}$	0.075
$Shear\;Modulus,\;G_{12}/\mathrm{GPa}$	6	$Density,\rho /\left(t/mm^{3}\right)$	1.69
$Shear\;Modulus,\;G_{13}/\mathrm{GPa}$	6		
$Shear\;Modulus,\;G_{23}/\mathrm{GPa}$	3.7		

**Table 2:** Material properties of Araldite 2015 epoxy adhesive.

Properties	Value
Young’s modulus, E/GPa	2
Shear modulus, G/GPa	0.9
Shear modulus, G/GPa	30
$Compressive\;strength,\;\tau_{s}^{0}/\mathrm{GPa}$	0.014
$Shear\;strength,\tau_{t}^{0}/\mathrm{GPa}$	0.014

**Table 3:** Parameters of control experiments.

Reference Experiment	*L*_1_	*L*_2_	*C*_1_	*C*_2_
a	90	30	5	5
b	90	30	5.3	5
c	90	15	5	5
d	60	15	5	5

**Table 4:** The impact of the sample amount on the accuracy of ultimate failure force prediction.

Sample Amount	100	200	300	400	500
Training set accuracy	97.5%	97.9%	99.2%	99.5%	99.6%
Testing set accuracy	91.5%	92.6%	97.5%	98.9%	99.2%

**Table 5:** Ultimate failure force error distribution.

Error	0–0.5%	0.5–1%	1–1.5%	1.5–2%	2–2.5%
Training set	66%	23%	3%	3%	2%
Testing set	56%	26%	4%	4%	2%

## Data Availability

The data that support the findings of this study are available from the corresponding authors upon request.

## References

[1] Rajak D., Pagar D., Menezes P., Linul E. (2019). Fiber-Reinforced Polymer Composites: Manufacturing, Properties, and Applications. Polymers.

[2] Yao S.-S., Jin F.-L., Rhee K.Y., Hui D., Park S.-J. (2018). Recent Advances in Carbon-Fiber-Reinforced Thermoplastic Composites: A Review. Compos. Part B Eng..

[3] Swolfs Y., Gorbatikh L., Verpoest I. (2014). Fibre Hybridisation in Polymer Composites: A Review. Compos. Part A Appl. Sci. Manuf..

[4] Amor N., Noman M.T., Petru M. (2021). Classification of Textile Polymer Composites: Recent Trends and Challenges. Polymers.

[5] Liu Y., Zwingmann B., Schlaich M. (2015). Carbon Fiber Reinforced Polymer for Cable Structures—A Review. Polymers.

[6] Pawlak A.M., Górny T., Dopierała Ł., Paczos P. (2022). The Use of CFRP for Structural Supervised—Literature Review. Metals.

[7] Lima R.A.A., Tao R., Bernasconi A., Carboni M., Carrere N., Teixeira De Freitas S. (2023). Uncovering the Toughening Mechanisms of Bonded Joints through Tailored CFRP Layup. Compos. Part B Eng..

[8] Zhang J., Lin G., Vaidya U., Wang H. (2023). Past, Present and Future Prospective of Global Carbon Fibre Composite Developments and Applications. Compos. Part B Eng..

[9] Sun G., Yu H., Wang Z., Xiao Z., Li Q. (2019). Energy Absorption Mechanics and Design Optimization of CFRP/Aluminium Hybrid Structures for Transverse Loading. Int. J. Mech. Sci..

[10] Wang Y., Zhang T., He Y., Ye J., Zhang H., Fan X. (2022). Analysis of Damage of Typical Composite/Metal Connecting Structure in Aircraft under the Influences of High-Velocity Fragments. Appl. Sci..

[11] Liu K., Liu Y., Sabbrojjaman M., Tafsirojjaman T. (2023). Effect of Bolt Size on the Bearing Strength of Bolt-Connected Orthotropic CFRP Laminate. Polym. Test..

[12] Xu C., Wang W., Liu Z., Fu C. (2020). Prediction Model and Parametric Study on CFRP Flat-Joggle-Flat Hybrid (Bonded/Bolted) Joints. J. Compos. Mater..

[13] Gamdani F., Boukhili R., Vadean A. (2019). Tensile Behavior of Hybrid Multi-Bolted/Bonded Joints in Composite Laminates. Int. J. Adhes. Adhes..

[14] Gamdani F., Boukhili R., Vadean A. (2022). Fatigue Behavior of Hybrid Multi-Bolted-Bonded Single-Lap Joints in Woven Composite Plates. Int. J. Fatigue.

[15] Ulus H. (2022). An Experimental Assessment of Hybrid Bolted/Bonded Basalt Fiber Reinforced Polymer Composite Joints’ Temperature-Dependent Mechanical Performances by Static and Dynamic Mechanical Analyses. Int. J. Adhes. Adhes..

[16] Jiang L., Dong D., Xiao S., Chen D., Yang B., Yang G., Zhu T. (2022). Experiment and Simulation Study on Bonded, Bolted and Hybrid Bolted/Bonded Joints of Textile CFRP Using Bimodulus Constitutive Model. Int. J. Adhes. Adhes..

[17] Zheng Y., Zhang C., Tie Y., Wang X., Li M. (2022). Tensile Properties Analysis of CFRP-Titanium Plate Multi-Bolt Hybrid Joints. Chin. J. Aeronaut..

[18] He B. (2020). Experimental and Semianalytical Investigation of X850 ± IM190 CFRP Bolted Joints. Adv. Compos. Lett..

[19] Li X., Xu B., Hong Y., Luo H. (2023). A Detailed Experimental Parametric Analysis of Bolted and Hybrid Bolted/Bonded Composite Joints. J Appl. Polym. Sci.

[20] Zhang Y., Sun Y. (2021). Analysis of Effects of Polyhedral Oligomeric Silsesquioxane on Thermal Properties of Epoxy Resin. Eng. Plast. Appl..

[21] Zhang Y., Sun Y. (2022). Study on Atomic Oxygen Resistance of EP Resin Modified by POSS. New Chem. Mater..

[22] Delzendehrooy F., Akhavan-Safar A., Barbosa A.Q., Carbas R.J.C., Marques E.A.S., Da Silva L.F.M. (2022). Investigation of the Mechanical Performance of Hybrid Bolted-Bonded Joints Subjected to Different Ageing Conditions: Effect of Geometrical Parameters and Bolt Size. J. Adv. Join. Process..

[23] Li X., Cheng X., Guo X., Liu S., Wang Z. (2020). Tensile Properties of a Hybrid Bonded/Bolted Joint: Parameter Study. Compos. Struct..

[24] Xu Z.X. (2014). FE Modeling for Load Distribution Analysis of Multi-Bolt Composite Joints. AMM.

[25] Liu F., Zhang J., Zhao L., Xin A., Zhou L. (2015). An Analytical Joint Stiffness Model for Load Transfer Analysis in Highly Torqued Multi-Bolt Composite Joints with Clearances. Compos. Struct..

[26] Liu F., Lu X., Zhao L., Zhang J., Hu N., Xu J. (2018). An Interpretation of the Load Distributions in Highly Torqued Single-Lap Composite Bolted Joints with Bolt-Hole Clearances. Compos. Part B Eng..

[27] Qiu C., Han Y., Shanmugam L., Jiang F., Guan Z., Du S., Yang J. (2022). An Even-Load-Distribution Design for Composite Bolted Joints Using a Novel Circuit Model and Neural Network. Compos. Struct..

[28] McCarthy M.A., McCarthy C.T., Padhi G.S. (2006). A Simple Method for Determining the Effects of Bolt–Hole Clearance on Load Distribution in Single-Column Multi-Bolt Composite Joints. Compos. Struct..

[29] Gu Z., Liu Y., Hughes D.J., Ye J., Hou X. (2021). A Parametric Study of Adhesive Bonded Joints with Composite Material Using Black-Box and Grey-Box Machine Learning Methods: Deep Neuron Networks and Genetic Programming. Compos. Part B Eng..

[30] Shan Libin Zhao M., Huang W., Liu F., Zhang J. (2020). Effect Mechanisms of Hygrothermal Environments on Failure of Single-Lap and Double-Lap CFRP-Aluminum Bolted Joints. Comput. Model. Eng. Sci..

[31] Guo K., Yang Z., Yu C.-H., Buehler M.J. (2021). Artificial Intelligence and Machine Learning in Design of Mechanical Materials. Mater. Horiz..

[32] Wang H., Duan Y., Abulizi D., Zhang X. (2017). Design Optimization of CFRP Stacking Sequence Using a Multi-Island Genetic Algorithms under Low-Velocity Impact Loads. J. Wuhan Univ. Technol. Mat. Sci. Edit..

[33] (2013). Test Method for Bearing Response of Polymer Matrix Composite Laminates.

[34] Hou Y., Wang W., Meng L., Sapanathan T., Li J., Xu Y. (2022). An Insight into the Mechanical Behavior of Adhesively Bonded Plain-Woven-Composite Joints Using Multiscale Modeling. Int. J. Mech. Sci..

[35] Shi J., Yang X., Chen X., Du K., Li C., Yang Y. (2024). Numerical and Experimental Investigation on the Load-bearing Performance of Plain-woven Composites Hybrid Bonded-bolted Joints. Polym. Compos..

[36] Tedjopurnomo D.A., Bao Z., Zheng B., Choudhury F., Qin A.K. (2020). A Survey on Modern Deep Neural Network for Traffic Prediction: Trends, Methods and Challenges. IEEE Trans. Knowl. Data Eng..

[37] Nafees A., Amin M.N., Khan K., Nazir K., Ali M., Javed M.F., Aslam F., Musarat M.A., Vatin N.I. (2021). Modeling of Mechanical Properties of Silica Fume-Based Green Concrete Using Machine Learning Techniques. Polymers.

[38] Xue X., Makota C., Khalaf O.I., Jayabalan J., Samui P., Abdulsahib G.M. (2023). Machine Learning Approach for Prediction of Lateral Confinement Coefficient of CFRP-Wrapped RC Columns. Symmetry.

[39] Singhal S., Jatana N., Subahi A.F., Gupta C., Ibrahim Khalaf O., Alotaibi Y. (2023). Fault Coverage-Based Test Case Prioritization and Selection Using African Buffalo Optimization. Comput. Mater. Contin..

[40] Rahman H., Tariq J., Ali Masood M., Subahi A.F., Ibrahim Khalaf O., Alotaibi Y. (2023). Multi-Tier Sentiment Analysis of Social Media Text Using Supervised Machine Learning. Comput. Mater. Contin..

[41] Banumathy D., Ibrahim Khalaf O., Andr閟 Tavera Romero C., Vishnu Raja P., Kumar Sharma D. (2023). Breast Calcifications and Histopathological Analysis on Tumour Detection by CNN. Comput. Syst. Sci. Eng..

[42] Chang Z., Zhang Y., Chen W. (2019). Electricity Price Prediction Based on Hybrid Model of Adam Optimized LSTM Neural Network and Wavelet Transform. Energy.

[43] Smith L.N. (2017). Cyclical Learning Rates for Training Neural Networks. Proceedings of the 2017 IEEE Winter Conference on Applications of Computer Vision (WACV).

[44] Srivastava N., Hinton G., Krizhevsky A., Sutskever I., Salakhutdinov R. (2014). Dropout: A Simple Way to Prevent Neural Networks from Overfitting. J. Mach. Learn. Res..

[45] Liu X., Qin J., Zhao K., Featherston C.A., Kennedy D., Jing Y., Yang G. (2023). Design Optimization of Laminated Composite Structures Using Artificial Neural Network and Genetic Algorithm. Compos. Struct..

